# Patient experiences of ART adherence clubs in Khayelitsha and Gugulethu, Cape Town, South Africa: A qualitative study

**DOI:** 10.1371/journal.pone.0218340

**Published:** 2019-06-20

**Authors:** Emilie Venables, Catriona Towriss, Zanele Rini, Xoliswa Nxiba, Tali Cassidy, Sindiso Tutu, Anna Grimsrud, Landon Myer, Lynne Wilkinson

**Affiliations:** 1 Southern Africa Medical Unit, Médecins Sans Frontières, Cape Town, South Africa; 2 Division of Social and Behavioural Sciences, School of Public Health & Family Medicine, University of Cape Town, Cape Town, South Africa; 3 Centre for Actuarial Research, Faculty of Commerce, University of Cape Town, Cape Town, South Africa; 4 Division of Epidemiology & Biostatistics, School of Public Health & Family Medicine, University of Cape Town, Cape Town, South Africa; 5 Médecins Sans Frontières, Khayelitsha, South Africa; 6 Division of Public Health Medicine, Faculty of Health Sciences, University of Cape Town, Cape Town, South Africa; 7 Western Cape Government Health Department, Cape Town, South Africa; 8 International AIDS Society, Cape Town, South Africa; 9 Centre for Infectious Disease Epidemiology & Research, School of Public Health & Family Medicine, University of Cape Town, Cape Town, South Africa; University of North Carolina at Chapel Hill, UNITED STATES

## Abstract

**Background:**

Globally, 37 million people are in need of lifelong antiretroviral treatment (ART). With the continual increase in the number of people living with HIV starting ART and the need for life-long retention and adherence, increasing attention is being paid to differentiated service delivery (DSD), such as adherence clubs. Adherence clubs are groups of 25–30 stable ART patients who meet five times per year at their clinic or a community location and are facilitated by a lay health-care worker who distributes pre-packed ART. This qualitative study explores patient experiences of clubs in two sites in Cape Town, South Africa.

**Methods:**

A total of 144 participants took part in 11 focus group discussions (FGDs) and 56 in-depth interviews in the informal settlements of Khayelitsha and Gugulethu in Cape Town, South Africa. Participants included current club members, stable patients who had never joined a club and club members referred back to clinician-led facility-based standard care. FGDs and interviews were conducted in isiXhosa, translated and transcribed into English, entered into NVivo, coded and thematically analysed.

**Results:**

The main themes were 1) understanding and knowledge of clubs; 2) understanding of and barriers to enrolment; 3) perceived benefits and 4) perceived disadvantages of the clubs. Participants viewed membership as an achievement and considered returning to clinician-led care a ‘failure’. Moving between clubs and the clinic created frustration and broke down trust in the health-care system.

**Conclusions:**

Adherence clubs were appreciated by patients, particularly time-saving in relation to flexible ART collection. Improved patient understanding of enrolment processes, eligibility and referral criteria and the role of clinical oversight is essential for building relationships with health-care workers and trust in the health-care system.

## Introduction

Globally, 37 million people are in need of lifelong ART with 21.7 million accessing treatment in 2016 [1]. Acceptance and increasing implementation of the ‘Treat All’ approach to anti-retroviral (ART) provision has changed the emphasis from ‘who’ is eligible to start ART and ‘when’, to ‘how’ to provide ART care and drug delivery to all HIV-infected patients [1]. With the continual increase in the number of patients starting ART and the need for life-long retention and adherence, increasing attention is being paid to differentiated service delivery (DSD) across the HIV cascade to better serve the diverse needs of people living with HIV [2]. DSD initially focused on designing and implementing ART delivery models that made access to ART care and drug refills simpler for clinically stable people living with HIV.

Reported retention and viral suppression outcomes have suggested that differentiated models of ART delivery better serve the needs of clinically stable patients, but to fully understand whether models have met this stated purpose and whether adjustments could be made to further improve the patient’s HIV care, an in-depth exploration of the patient experience is required [3–9]. Differentiated ART delivery can only significantly improve cohort level retention and viral suppression if implemented at scale. Studies reporting outcomes of differentiated ART delivery at scale have called for a better understanding of both health system and patient barriers to participation [7, 10].

While DSD primarily focuses on three elements: clinical characteristics; sub-population type and context [2], many patients will likely shift between some of these categorisations over a lifetime of treatment. These shifts may include transitioning from youth to adulthood, pregnancy and parenthood, periodic changes between rural and urban contexts and importantly, periods of viral rebound. The extent to which differentiated ART delivery models support these transitions without complicating care necessitates consideration.

Several sub-Saharan African countries including Uganda, Kenya, the Democratic Republic of Congo, Malawi and Mozambique have implemented differentiated models of ART delivery [4, 8–11]. The ART adherence club (AC) model is a healthcare worker managed group model designed to meet the needs of clinically stable adult ART patients. In the Western Cape province of South Africa, the AC model was initially piloted by Médecins Sans Frontières (MSF) and the Western Cape Department of Health (WCDoH) and subsequently adopted by the WCDoH and implemented across the entire Cape Town health district [12]. At scale, patient level outcomes of retention and viral suppression are high [12]. In a cluster random sample of 10% of the Cape Town ACs (3,216 adults) from non-research supported ART sites, retention was 95% at 12 months and 89% at 24 months. In the 13 months prior to database closure, 88% of clients had viral loads (VL) taken with VL ≤400 copies/ml in 97% [6]. An evaluation of disengagement from care in Khayelitsha, Cape Town reported AC participation as highly protective against disengagement [13].

While there have been qualitative studies exploring patient perceptions of other models of differentiated ART delivery, to date there has been no qualitative exploration of people’s experiences of the AC model in South Africa or of the experiences when a model is at scale, other than to inform a recent mixed methods health system analysis [14–19]. Using qualitative methods, we explored perceptions of ACs among former and current AC members, as well as those who had never joined a club, in two settings in Cape Town, South Africa, including the perceived advantages and disadvantages of the differentiated model mechanisms. We also explored the experiences of patients referred out of ACs back to routine clinician-led facility-based care and patient perceptions regarding barriers to AC participation.

## Methods

### Study setting

The Cape Metro health district serves a largely urban population of approximately 3.75 million people in and around Cape Town in the Western Cape province of South Africa [19]. The antenatal HIV prevalence in the district was 21.7% in 2013, compared to a national average of 29.7% [20]. The clinics included in this study are two of the largest public-sector service sites. The Ubuntu ART clinic at Site B community health centre (CHC) in Khayelitsha and Gugulethu CHC had 9,904 and 5,666 patients retained in care at the end December 2016, respectively. Ubuntu was the pilot site for ACs and Gugulethu was one of the first CHCs to implement the model. At the time of the study, Khayelitsha had 4,553 patients (46% of their ART cohort) in ACs and Gugulethu had 2,718 (48%). Both sites have previously reported quantitative outcomes [3, 21]. Both communities have high work related migration between the Western Cape and Eastern Cape provinces with patients frequently travelling back home to the Eastern Cape, especially during holiday periods.

### Description of the AC model in Khayelitsha and Gugulethu

The AC model has been described in detail elsewhere [10, 13, 22, 23]. In summary, the model provides ART drug distribution, HIV/ART clinical management and peer-support to groups of clinically stable, HIV positive adult patients. Patients are eligible for ACs if they have been on ART for six months, have an undetectable viral load result (<400 copies/mL) and no clinical condition requiring more frequent clinical follow-up. Patients on second line ART are also eligible for club membership.

ACs are comprised of 25–30 patients who meet with a lay health-care worker five times a year (a four month ART supply is provided over the December holiday period when many people return to the Eastern Cape) for 30–60 minutes for a short symptom screen, peer support and distribution of pre-packed ART. The lay facilitator of the club carries out the brief symptom check at each club visit and a nurse is responsible for the blood draw for viral load at the annual clinical consultation. Psycho-social support includes a facilitated group discussion led by the lay health-care worker and peer support from other group members. The lay health-care worker is also available to provide individual support to members before or after the group if required. The guided discussion provides an opportunity to use the group session to discuss or provide information about a specific topic, such as a change in first or second line drug regimen. The length of time spent in guided discussions varies on the group and the facilitator, with some well-established and long running clubs preferring to reduce the length of discussion time in favour of a quick medication pick-up.

Some ACs are facility-based, while others have been decentralised to community venues and patient homes. Blood is drawn for viral load monitoring by a professional nurse at the second AC visit for all AC members, with each attending an individual clinical consultation at the third AC visit. This schedule repeats annually. If symptomatic at any AC visit, AC patients have priority access to a designated facility nurse. AC patients can also attend the clinic at any other time should they have clinical complaints. If a patient experiences viral rebound in the AC, fails to attend their AC within five days of the AC meeting date (the ‘grace period’) or becomes clinically unstable for any reason which requires closer clinical follow-up, the patient is referred out of the AC back into clinician-led facility-based care for ongoing management and enhanced support. They can then return to a club at the discretion of a clinician: in such cases, they do not need to meet the eligibility criteria again, but require an undetectable viral load if it was previously elevated. Patients can also ask a ‘buddy’ such as a relative or friend to attend the meeting on their behalf (referred to as the ‘buddy system’) and pick up their medication every alternate AC visit.

While Khayelitsha and Gugulethu follow the same AC model described above, the two sites have different club locations available to clients. At the Ubuntu Clinic in Khayelitsha, clients have a choice between four Adherence Club locations: i) Facility-based clubs which take place in a room in the clinic ii) Community clubs close to the facility which take place at a room adjacent to the local library 500m from Ubuntu ART clinic iii) Community clubs which take place in various community venues such as a hall or church one to two kilometres away from the patient’s home and iv) home clubs, which take place in a one of the AC member’s homes and have fewer members (eight to 12). Community clubs and home clubs are not available in all feeder areas. At the Gugulethu CHC in Gugulethu, all clubs take place in the same community venue approximately 1km from the clinic.

### Study recruitment and sampling

Participants were recruited from the two clinic sites using purposive sampling. Potential participants were contacted about the study both in person and telephonically and told that the study was being conducted to learn more about people’s experiences in clubs. Current AC members, patients who had never joined an AC despite being eligible and patients who had been referred out of club care for non-attendance or clinical reasons including viral rebound, were included in the study. Those not in clubs were recruited from the clinic waiting rooms after their eligibility for the study was checked in their clinical folder. Patients who were no longer in clubs were identified from club registers as having been referred back to the clinic or lost to follow up from the club and then contacted telephonically to be invited to participate. FGDs were composed of club members who were informed about the study and recruited during their regular club sessions.

Additional effort was made during recruitment to ensure that men were included in the sample, but recruitment of male participants was challenging as many had employment commitments and were not available thus refused participation. In addition, AC members were sampled to include those in facility-, home- and community-based clubs.

In-depth interviews (IDIs) were conducted with patients who were not in ACs, which included i) AC eligible patients currently in clinic care who have never participated in an AC, ii) those referred out of an AC due to non-attendance and iii) those referred out of an AC due to virological rebound. In addition, one IDI was conducted with an AC member who was unable to attend the scheduled focus group discussion (FGD) but still wanted to participate in the study. FGDs were conducted with current AC members, as these were people who were comfortable engaging in a group and we wanted to learn more about the group dynamics of ACs from them, whereas IDIs were conducted with those who had individual experiences of non-participation, non-attendance, virological failure or loss to follow-up that we wanted to discuss with them.

### Data collection

In 2016, IDIs and FGDs were conducted in isiXhosa by two local, female research assistants trained in qualitative research methods. Neither the research assistants nor the PI worked in the clinics where the research took place or had a prior relationship with the participants before the study began. The researchers explained to potential participants that the study was being conducted to learn more about the clubs, and people’s experiences of them. Interview and FGD guides were developed in English before being translated into isiXhosa. Guides focussed on people’s experiences and preferences relating to ACs, including any challenges and benefits of the model and were piloted before the study began. Initial FGDs were supported by the female PI, who has a PhD in the field of social sciences and was working for MSF at the time of the study, but no-one else was present during the remaining interviews or FGDs. A total of 11 FGDs with 88 participants were conducted in the two study sites (Table 1). IDIs and FGDs mostly took place in the MSF office in Khayelitsha and in a community venue in Gugulethu, which was selected as a place that participants were familiar with. FGDs were not divided by gender, as AC members are used to mixed-gender group discussions during their regular sessions. In addition, 56 IDIs were conducted with participants from the categories outlined below (Table 2).

**Table 1:** Categories of club members participating in focus group discussions (FGDs).

Category of club member*	Male	Female	Total FGD participants
Facility club	2**	20	22
Community club: Close to facility***	13	32	45
Community club: Close to patient’s home	1	13	14
Home club	3	4	7
Total	19	69	88

* Data on gender and type of club is unavailable for two club members

**1 male participant belonged to a male only facility club

***500m-1km from patient’s ART facility

**Table 2:** In-depth interview participants.

Category of interviewee	Male	Female
Eligible but never attended a club	4	21
Referred back: high viral load	3	13
Referred back: non-attendance	2	11
Referred back: unknown reason	0	1
Community club member	0	1
Total	9	47

Individuals only took part in one IDI or FGD. Whilst saturation was reached in some categories, due to difficulties in recruiting those who had disengaged from care, men and younger participants, saturation was not reached across all categories of participant. The numbers of potential participants who were unable to take part were not systematically documented, but the main reasons for non-participation were other commitments at home or work. The mean duration for the FGDs was 143 minutes, and the mean duration for the IDIs was 32 minutes.

### Data analysis

All FGDs and IDIs were audio-recorded before being transcribed and translated from isiXhosa into English and notes were also taken during data collection. All transcripts were entered into an NVivo database (NVivo qualitative data analysis Software; QSR International Pty Ltd. Version 10, 2012) and were coded by the PI using both inductive and deductive coding. A thematic analysis approach was applied. Codes and emerging themes were discussed with other co-investigators to ensure the validity of findings and to discuss any discrepancies and the main themes are presented below in the results.

### Ethics approvals

This research was granted ethics approval by the Human Research Ethics Committee of the University of Cape Town, Faculty of Health Sciences, and permission was sought from the Provincial Government of the Western Cape Department of Health. Written informed consent, administered in isiXhosa, was obtained from all participants before beginning data collection.

The findings of this study have been reported following the Consolidated Criteria for Reporting Qualitative Research (COREQ) guidelines [24].

## Results

Of the 144 participants, 28 were male and 116 were female. Twenty-five interviewees had never attended a club; 30 had been referred-back to the clinic for viral rebound, poor attendance or other reasons and the remaining participants were all members of home, community or facility clubs. Interviewees ranged in age from 25 to 67, with a mean age of 41 years.

The main themes identified during coding were i) people’s understanding and knowledge of the AC mechanism; ii) understanding of and barriers to enrolment; iii) perceived benefits including time-saving, the buddy system, the grace period, peer support, reduced stigma and the benefits of different kinds of clubs; and iv) perceived disadvantages of the AC, particularly referral back to clinician-led ART facility-based care (“standard care”). In total, 93 codes were developed and these were refined into the four broad themes listed above.

### Knowledge of ACs: *“They say they do not stay long.”*

Overall, AC members and non-members had a good knowledge of ACs and how they work, but there was some confusion in both clinics about the ongoing eligibility criteria for ACs as well as referral-back procedures. Participants heard about ACs from peer education sessions in the clinic waiting rooms, the Treatment Action Campaign (a South African civil society organisation campaigning for access to health-care), their friends and other community members and from clinic staff. Many participants described how AC membership was determined by the length of time they had been on ART, their viral load and whether they kept their appointment dates. Others knew that there were exclusion criteria related to other conditions, such as TB, diabetes and hypertension.

One participant in an FGD in Khayelitsha described eligible patients as those who are ‘*doing well*, *taking your treatment well*, *coming to your appointments properly*’. Another patient who had previously been a member of an AC in Gugulethu before being referred back to standard care for non-attendance described eligibility criteria in the following way:

[T]he club is for people who are positive, who have known about their status for a while. It’s [for] people that know they are HIV positive and are living a positive life, that look healthy. It’s for people that are taking their pills properly and when their blood is checked their viral load is low… Those people can be taken immediately to the club because they are trusted to look after themselves unlike those that are beginners and the ones that are very weak.

There were some misunderstandings and lack of knowledge about who was eligible or who ‘qualified’ to join a club, and there was also uncertainty from non-club members about how to access and join an AC, as discussed below in more detail.

### Barriers to enrolment: *“They will decide whether you get taken there*.*”*

Current and former AC members, and those who had never joined a club were asked about their knowledge and experience of the AC enrolment procedures.

Reasons for not joining ACs related to a lack of knowledge about AC eligibility and recruitment procedures, practical reasons including timing (ACs during work hours) and preferring what was perceived to be the enhanced medical care provided in standard care. AC members emphasised that joining a club was a choice, but some non-members did not have enough information about the model to be able to make this decision themselves.

When current and previous AC members described how they were enrolled, most talked about a health-care provider who initiated the conversation, or explained that they were *told* to join. Similarly, non-AC members described feeling unable to ask healthcare workers about joining an AC instead of waiting for it to be suggested to them. Whilst a few interviewees said that they had initiated the enrolment process, most, such as the female patient cited below, had waited to be asked or felt that clinicians and nurses were too busy to answer their questions:

When I asked them [about clubs] they said that “we [nurses] are always in a hurry”. We [patients] don’t sit down and enquire about things related to the club, even if it is something that can help us.

Several female patients including this non-AC member from Gugulethu explained how they had heard about clubs from a friend, but were not then offered the choice to enrol:

I always hear people say that if you take your pills well you move to [the club] and then I don’t know what happens then. I heard that it is for people who take their treatment well and in some the virus is invisible. I have been taking my pills. I have been taking them so the virus is no longer visible; I heard that in that case then you get transferred to the club, but what happens from there…I really don’t know. I never got to hear what happens from then onwards.

Another clinically stable patient who had never joined an AC stated multiple reasons for wanting to stay in the standard care, including wanting a doctor’s certificate to explain her absence to her employer, and not wanting to disclose her status to others:

[You don’t get a] doctor’s certificate, you don’t get it. When you are in a club you are in a club, and yes it is nice that you can send anyone, but… Some people don’t have the luxury of skipping work anytime and just phoning to say they won’t be coming in today. It’s not easy to disclose to people so if I don’t want to miss work I will be forced to send someone in my place, and this person will know why she is going to this place for me. It’s not easy to disclose, even to my family members, that I am like this because you don’t know what they might think, how they are going to react and so on. I would rather stay at the clinic so that if I miss a day I know I have a doctor’s certificate and there’s nothing I will have to argue about.

As the previous participant mentioned, another challenge with recruitment to ACs was the perception from patients that they would receive reduced clinical care by not seeing a clinician as frequently. This female patient who initiated ART in 2009 did not want to join an AC because she was concerned about reduced clinical support:

The disadvantages would be if you get there and you take the pills and go. You do not have time to talk to the nurse or doctor. Now [in the clinic], when I go take my pills if I feel something is wrong I can always talk about it as well.

Another female participant, who had been a member of an AC in Gugulethu before experiencing virological failure, was concerned about the effect limited clinical support could have on other patients:

It is not good for quiet people because you could die while taking pills without consulting a doctor. You see, at the club pills are not counted. You do not go there with your remaining pills, you go there with only your [clinic] card and get new pills. Some people can get sick from that because they do not take their pills. If you are in the club no one counts your pills or cares about you.

Her words were supported by another former AC member from Khayelitsha:

I don’t think it’s good because they sometimes do things that they think are right without consulting a doctor. I don’t think that’s the right way of doing things. It would be better if they did things with the doctor, and if the doctor said that this is the medication you should take.

### Perceived benefits

The perceived benefits of ACs included saving time, the buddy system, the grace period, peer support and reduced stigma.

**Saving time: “*You do not stay long*.”** The time-saving aspect of the ACs was one of the main benefits described by members, including this female patient in Khayelitsha who attended an AC in her local community:

It’s about taking services closer to people rather than waking up and going to a clinic that is far away. It’s about making a clinic nearer to where you stay. You can even wake up at 10:00 when your body is not feeling well, go and fetch your stuff from the clinic and come back home to have your porridge.

Time saving was also important to this community club member from Gugulethu:

You don’t spend the whole day in the clinic. It’s only 30 minutes to take your medication, then you go home.

AC members also appreciated the time saved by not having to queue in the clinic waiting area or at the clinic pharmacy, as they did not have to request time off from work or disclose their HIV status to their employers to explain their absence. Although there were some complaints about clubs sometimes starting late, the AC model—in and outside of facilities—was on the whole considered preferential to queuing in a clinic for several hours.

An additional benefit of ACs that were in people’s homes or local communities within a kilometre of their home was that members benefitted financially as they did not need to spend money on transport to attend.

**Buddy system: “*The thing I love most is that you can send somebody*.”** The buddy system was one of the most-valued aspects of AC membership, as patients such as this female member of a community club, who used a buddy to collect their medication, did not have to attend all of their AC meetings, described:

I like the community club because if you aren’t going to make it, you can give your neighbour your card so that she can go take your treatment. I like it that way.

This 34-year-old female left the AC because of virological rebound found the buddy system one of the main benefits of club membership:

The thing I love most is that you can send somebody and you don’t stay for a long time. People are too lazy to go to the clinic so I will tell her that she can ask her child, her husband or a person of her choice to go for her. It is not a must to go there by yourself. That is what is nice about the club.

This female interviewee from Gugulethu discussed how the buddy system benefitted her when she was travelling to the Eastern Cape and was unable to attend the clinic to collect her medication:

I can send someone else, like when I was working in East London [in Eastern Cape]. I told them that I will have my treatment support [buddy] who will be fetching my medication. So I sent my sister to go and collect my pills, so then whenever my [appointment] date arrives and I am still in East London she can go and fetch my medication. I was only being called when I have to draw blood to check how everything is.

Whilst AC members, such as this female member of a closer to home community club, appreciated the buddy system, there was also a sense that it was important to follow the ‘rules’ and not send someone every time:

When a person sends someone to fetch her treatment it must be for one month, not two or three months. It must only be for one month.

There was, however, one member of the same club who did not want to use the buddy system, as she wasn’t sure she could trust a buddy and she valued experiencing the club herself alongside the peer support it offered:

There are also times where we give others our cards, but they took a long time because they didn’t think it was important. Someone says they will do you a favour and she ends up going but doesn’t get the medication. She comes up with stories of not getting it and you end up not taking your treatment properly… There’s nothing that is boring there, it’s nice. We have time with the counsellors and we get used to them: you can talk about the changes that happen to you with this treatment. You can be open and not afraid of anything, but in the clinic they rush to somewhere, like lunch times, and you don’t have a chance to talk about what happens in your life. Here we get a chance to talk with others and you hear their experiences then you get better.

Other club members in Gugulethu and Khayelitsha talked about who their buddies were and why they had chosen them:

The person I make my buddy? I love a person who talks, who expresses herself, who is herself, who is free to talk. I don’t trust someone who is silent…even when we are talking she is silent…you don’t know what she is thinking, or if she is taking anything in.

This 44-year-old woman also discussed who can be a buddy:

Maybe your relative in the house or your trusted friend who knows that you are HIV positive, so they know everything. That is the person you can send to fetch your pills.

Some club members, such as this one also sent their children to collect their medication for them:

So when it is close I can even send my child to get me my pills at the club. I don’t have to request time off from work because even requesting time off from work gives you bad luck. Then it has to be your child and you need to trust them to fetch them for you. “*Go my child*”…maybe you have forgotten the card [so you say] “*Go and get the card there*, *and ask for the sister*, *show her the card and tell her I have sent you*”. That is how it helps us.

One former AC member from Khayelitsha described how she could also ask other members to collect her medication for her:

In the club we are like sisters and brothers so you can contact someone when they go there and ask them to fetch your medication. If you are working, you are working [and cannot come]. The club is helpful in that way. There is no risk of losing your job because at least you save time and can send somebody. The thing I like is that you can send someone.

One stable female patient who had not joined a club explained her feelings on sending a buddy:

The clubs, the thing that I know is that it is for people who are lazy to go to the clinic. They are not able to come to the clinic so then they join the club and in the club it becomes easy to ask someone to fetch your stuff.

**Grace period: *“Sisi*, *please keep my pills*.”** The grace period was also viewed as a benefit as it further reduced the amount of time patients needed to spend in the clinic and allowed AC members an additional flexibility with their appointment date and time. Many interviewees regularly used the grace period and saw it as one of the key advantages to joining an AC.

This patient had been referred back to the clinic and was no longer in an AC, but reflected on why she appreciated the grace period previously available to her:

What is very interesting in the club is that you can call and say that you are not going to make it to the club at a certain time you can only make it at a certain day, so “*please keep my pills*, *I will come and take them*”. The club is better than the clinic because at the clinic you cannot call, you have to go on your date.

Whilst many patients saw the grace period as a benefit, this 28-year-old female patient who had been referred out of an AC because of virological failure had not understood how the grace period operated:

The disadvantages of being at the club…for example there is a grace period. It was five days. I didn’t know that if you are not coming you will be taken out of the club and if your viral load becomes visible…you will go back to the chairs [the clinic waiting room]. And if you didn’t come in that five days and you are not sending a person you will have to go out of the club.

During the FGDs with club members there was, however, a sense that some people took advantage of the grace period to further reduce the time they spent collecting their medication.

**Peer support: *“You build that bond with people*.”** Peer support was also discussed by members and non-members as a benefit of the ACs. Some AC members described other club members as their ‘*family*’. This 28-year-old female community club member in Khayelitsha talked about the peer support offered by the AC, and how this differed from that given in the clinic:

[I]t is nice because you meet with people you don’t know and you end up having relationships with them. You receive the love you won’t get from people who are close to you. When you meet in the clinic you can’t talk about things because they call our names and people go to their places, but in the community club you sit down and chat and you know each other better.

Another female participant in the same FGD made a similar distinction between the peer support provided in the clinic queue and in their AC:

Sometimes you think something [a problem] is in you alone, but when you hear someone who has the same problem it’s good. At the clinic no one talks about their problems. Here we chat and you find others have bigger problems than yours and then you see yourself better. You see you are alive and not alone, and we share our problems when we are in the club. Here, we treat each other as a family: we are one family. I like it because the club helps us: in the clinic we don’t know each other and we don’t help each other.

Another way in which the peer-support provided by other AC members was seen to be beneficial was through the reduction of stigma. Many of the interviewees and FGD participants had experienced HIV-related stigma at home, in their communities or in the workplace, and were concerned about being discriminated against if they were seen at the clinic collecting their medication.

Patients in Gugulethu found the queue system particularly stigmatising because of how the patient files are labelled, believing that other patients could identify that they were HIV positive from looking at the colour of the sticker on their file. Belonging to an AC meant they did not have to feel stigmatised waiting in the clinic queue for several hours every month.

ACs were seen to reduce accidental disclosure and the possibility for people to be stigmatised, stigma because people were not waiting for long periods of time in a waiting room where they may be seen by others that they knew. The AC model also offered people peer-support from others living with HIV and coping strategies for dealing with stigma as people could share their experiences with each other. As this former AC member from Khayelitsha described, she knew very few people living with HIV before joining the club:

My problem is that I didn’t meet any people who had the virus so I knew nothing. The first time I took ARVs I knew nothing about it. There are some people who know [about my HIV status] but I’m not someone who talks to everyone.

However, one interviewee from Gugulethu explained that she was concerned about stigmatisation if she joined a club close to her home because of the gossip she had experienced in her community:

I thought that I cannot join a community thing. I know community things…you meet there in numbers. I used to stay in the shack, and in the shacks there is always gossip. It’s blah, blah, blah. I then thought I will never go to something with these people because I know how they are. You meet someone you know there and once she leaves the clinic [near to her home] they will spread to the entire section that you were there and it will be all that they talk about wherever they are. I did not go because of that. I stayed home.

### Perceived disadvantages

Most of the participants in the study were enthusiastic about ACs, with very few perceived disadvantages or drawbacks to membership. The main reported challenge was when patients were referred back to standard care for virological rebound or missed appointments and did not understand or accept the process of no longer being in an AC. There were also complaints from AC members about others who did not follow the ‘rules’ of the club and were perceived as taking advantage of the benefits that were offered to them.

**Referral-back mechanism: “*You are punished*.”** Study participants, whether currently or previously in clubs or never in an AC, perceived ACs as something which patients needed to ‘*qualify*’ for, and removal from a club was described as a ‘*failure*’ or punishment. Club members also distinguished themselves from ‘*the crowd*’ on the benches or chairs in the clinic waiting room, which FGD participants in Gugulethu in particular associated with being in poor health. AC membership was considered a privilege and returning to clinical care was considered undesirable

Being in a club is a great privilege because the treatment is good. If you are feeling sick and you go there, you just tell the nurse or the doctor that you are sick and they attend to you as soon as possible. Going to the club doesn’t come automatically: you have to work hard to be accepted in the club. If someone is doing well they will be promoted to go to a club.

Current and previous AC members, most of whom had no experience of being referred-back, did not want to return to standard care. Those who had experienced being referred-back did not enjoy the experience or want it to happen to them again, as returning to the waiting room was perceived as a punishment for not taking their treatment properly or for missing appointments.

One club member who was referred-back talked about not ‘*making the same mistake again’* and others described apologising for their ‘*mistakes’* again stressing the notion of a set of rules that should be followed. As an FGD participant from Khayelitsha stated:

If you don’t follow the rules, you will be dismissed… I was punished but I was reinstated [allowed to re-join the club later].

However, there were other participants who felt that this punishment was part of taking responsibility for their own health, including the responsibility for attending their AC appointments:

If you stay away for a month, that means you don’t care about your life. In the end, this [club] is meant to make your life easier… That shows that you don’t care because you should not make the mistake of going back to the clinic [required due to lateness] whilst already in the club.

Another participant in the same FGD felt that being in an AC was a privilege, but also believed that the threat of being asked to leave the club was ultimately beneficial to her:

It almost happened to me, but I didn’t see it as cruel. I saw it as a way to discipline me and educate me. It helped me to get the strength to disclose at work and tell them I have this problem [HIV]. I defaulted my appointment dates and did not come to the club, so I was shouted by the sister who told me that she would take me out of my group or give me a second chance. I said ‘give me the second chance’. So because of that, it sorted me. It’s [the ‘rules’] to help you stand up for yourself and know what you need in your life.

In addition, there was little understanding amongst either current or previous AC members of the purpose of referral-back. One interviewee from Khayelitsha described how after being referred-back, he had to attend the clinic and see the clinician more frequently. He considered the consultations a disadvantage as they took more time than being in an AC and did not see the value of increased clinical support.

**Experiencing standard care: “*Why did they have to take us aside*?”** AC members who had been referred-back from ACs to standard care felt frustrated and saw their situation as unfair, and in many cases blamed other people for their referral. An interview with a woman who was returned back to care from the AC because of virological failure described her situation:

It doesn’t make me happy because they removed me from the people I was always with… There is no need for them to remove us. Can’t they treat us while we are there with them? Why did they have to take us aside? Everyone looks at us because we are noticeable as those people whose virus increased.

This male patient who was referred back because of experiencing virological rebound had similar frustrations:

I love the club, but I am frustrated by this situation. I love the club. I liked the fact that I didn’t have to queue. You don’t queue when you are in the club, and you take your stuff and leave.

Some patients blamed the lay health-care workers facilitating the ACs, saying they had been treated unfairly or had not been given the correct information about their appointments which in turn caused them to miss AC sessions. An interviewee from Khayelitsha who had been referred back for missing appointments did not believe it was their fault:

I am not out of the club as such, but there was a misunderstanding about the date because of someone’s handwriting. The date was not clear. When you see a ‘5’ looking as if it is an ‘8’ and you find out that it is ‘5’… I also learnt a lesson while I am here. I need to be sure that the date I am given is really the date I see on the card.

One interview was with a participant who had disengaged from care after being referred back to the clinic. Despite saying that they would prefer to queue ‘*from bench to bench’* after a negative AC experience, upon being referred back, they did not return to clinical care at all. The interviewee described feeling despondent and stated “*I do not want anything to do with the club anymore*” stating that they would prefer waiting for several hours in the clinic instead.

Other reasons patients described referrals back to standard care as unfair were not responding to their treatment even if they were adherent, switching to second line treatment (although there are participants on second line treatment in some ACs), being diagnosed with another health condition such as diabetes, being hospitalised for another condition or being pregnant.

## Discussion

This qualitative study suggests that ACs are both acceptable and valued among people living with HIV in Gugulethu and Khayelitsha. The main gap in knowledge was around eligibility for enrolment and the criteria and rationale for patients to be referred back to standard care. Based on these findings and the perceived benefits and disadvantages of Adherence Clubs, recommendations for improving Adherence Clubs and suggestions for further research are outlined.

The results highlight the main patient-perceived benefit to AC membership is the flexibility it offers compared to standard care, and the way that patients can have differing levels of engagement with care. These valued flexibility benefits include time saving, enabling drug pick-up by a friend or relative through the buddy system, allowing drug pick-up for a period after the appointment date (the grace period), reduced transport costs when the drug pick-up point is located closer to home, peer support, convenience and a decrease in stigma. On the other hand, AC members can also reduce their personal engagement with the group on occasions that it is necessary. Similar benefits have been reported through qualitative studies conducted in other settings [14–17].

Members clearly see belonging to an AC as an achievement that comes with certain privileges, but these privileges can heighten the feelings of frustration and failure when referred back to care. Frustration was experienced by patients who did not understand the eligibility for joining and belonging to an AC, which would, in turn, allow access to these benefits. Frustration and feelings of failure and being punished were also experienced by patients who had been referred-back to standard care.

Overall, knowledge of the availability and working of clubs was good amongst all participants in the study, but there remained some confusion over recruitment processes and initial and continued eligibility criteria both among AC and non-AC patients. Patient understanding and buy-in to the AC model’s stated purpose, specifically the ongoing requirement to qualify as clinically stable which in turn allows for less intensive follow-up and simpler drug refill options, is important for the model to succeed for those who will not always be able to attend or remain adherent. Where there is limited buy-in, patients will likely not value returning to standard care for increased adherence and clinician attention in times of need. Patients may resent the process causing a breakdown of trust essential for the treatment of a disease that requires lifelong treatment and care or even worse, may choose not to return to standard care at all and be lost to follow up.

Many people living with HIV in Khayelitsha and Gugulethu still experience stigmatisation in their communities, and for some the club is a place where they feel able to share their worries and concerns with others in a similar situation. Clubs, similar to Community ART Groups (CAGs) in other contexts in Southern Africa, can provide a sense of community and peer support for many patients that is not felt in many clinic settings, as they see the same people regularly and meet in a private space away from the waiting room [16, 17]. For some patients, being in an AC also meant that they were less likely to need to disclose their HIV status to their employer, as they did not have to take as much time off work to attend clinic appointments, but the fear they still had about others finding out that they were HIV positive remains concerning.

Whilst AC members repeatedly stressed the importance of not missing appointments, being on time and adhering to treatment, there was little ownership from study participants over actions that may have led to them being referred back to standard care. Many patients blamed external factors including health-care workers or the health-care system itself when they were removed from ACs, suggesting that even though the model gave patients more autonomy, they were still within a health-care system where communication and relationships between patients and providers could be improved. Building trust between patients, the healthcare workers and the health-care system itself could help to improve understanding of the AC mechanisms and help to decrease frustrations with the eligibility criteria and the breakdown in trust that can occur when people are moved between ACs and routine care. Improved communication could also lead to increased patient empowerment, and may enable those who did not join a club because they felt unable to request it or have the opportunity to ask further questions.

If a club member is returned back to standard care, they are theoretically supposed to receive enhanced adherence and clinical support, but this was not always appreciated by the participants, leading us to question whether or not the package of care offered to ‘unstable’ patients who have been removed from ACs is appropriate or needs to be adapted. Where attention is not paid to the components and delivery of an enhanced package of care, patients may merely be returning to the long waiting times with very little counsellor or clinician contact.

As suggested by a former club member, an alternative option would be to allow people to stay in the club and add enhanced adherence and clinical support at the clinic until the patient is considered ‘stable’ again. This has been done elsewhere where the AC model was adapted for young people. In another clinic in Khayelitsha, young people are only returned to standard clinical care from the youth clubs after continued non-attendance or virological rebound and in a clinic in Johannesburg, South Africa, young patients remain in clubs remain there throughout management. This could be a way of reducing the frustrations felt by the adults in the study who had been referred out of care and may provide vital and enhanced peer support during a time of need [25, 26]. However, such an adaptation could create difficulties with those AC members who felt strongly that there should be clear, ongoing eligibility criteria for membership and who felt certain members abused the AC model flexibilities. It may also add an additional visit burden for those already struggling to meet appointments.

Based on our findings, further piloting and implementation research could be warranted. The results from the different groups of participants raise the question that it may be counter-productive to remove people from clubs even if they are not meeting the ongoing eligibility criteria. The benefits of being in a club, including the flexibility, peer support, reduction in stigma and ease of communicating with club staff, may in fact do more to assist patients who are struggling than referral-back does. Further research could focus on whether or not adding enhanced adherence support and clinical consultation alongside the AC model either as a brief intervention or until a patient is stable again could in fact be more beneficial. Studies such as this one enable us to interrogate the value of providing additional support within an AC rather than taking membership away. Assisting patients in ACs to become stable again instead of changing their service delivery model may reduce risk of losing patients to care and improve relationships with health-care staff and the healthcare system overall.

This is among the first qualitative studies exploring patient perceptions of a specific differentiated model of ART delivery at scale providing an essential addition to the quantitative outcomes analyses reported on to date. It is the only qualitative study to date that includes perspectives from patients who were eligible but did not join ACs or joined an AC and were referred-back to standard care for requiring more intensive follow-up. There are, however, some limitations to this study. One limitation is that participants may have assumed that the research assistant was a counsellor or health-care worker despite a thorough informed consent process, thus meaning patients were reluctant to criticise the AC model in fear of reprisals. Whilst additional effort was put into trying to recruit men for FGDs, this was not always possible due to their work commitments. As there were substantially more women in the club cohort overall, our FGDs had more female participants than male. The inclusion of more men in the study may have led to an increased focus on the need for flexible timing of clubs outside of standard working hours, and may have led to different perspectives on peer support. There were also limited younger adults included in the study meaning that we were unable to include their perspectives on ACs. In addition, some of the mixed gender FGDs had very vocal, dominant male participants who may have prevented others from voicing their opinions. It was extremely difficult to locate patients who were lost to care, and many of those contacted did not want to be interviewed, thus their perspectives are limited in this study and saturation was not reached amongst this group. In addition, we were unable to return the transcripts to participants for comments or correction, but ensured that they were discussed amongst the research team, particularly those conducting the interviews.

## Conclusions

ACs provide stable patients with a flexible solution to receiving ongoing HIV care, importantly including collecting their drug refills. Patients are curious about joining and feel privileged to belong to an AC, but this sense of privilege heightens the frustrations and sense of failure if a patient no longer qualifies as ‘stable’ and is referred-back to standard care, risking both short- and long- term retention. Improved patient understanding of recruitment as well as buy-in to the requirement to continuously qualify as stable, allowing for less intensive follow-up and simpler drug refill options, is important. This will particularly benefit patients during periods of clinical instability where more intensive follow-up is appropriate. Further research should determine whether increased adherence and clinical support alongside continued AC membership improves outcomes for these patients.
